# A Rare Case of Carpal Tunnel Syndrome Due to Subepineural Hematoma of Median Nerve: Diagnosis and Surgical Management

**DOI:** 10.7759/cureus.68201

**Published:** 2024-08-30

**Authors:** Christos Lyrtzis, Athina Stamati, Elvira Stathopoulou, George Paraskevas

**Affiliations:** 1 Anatomy and Surgical Anatomy, Aristotle University of Thessaloniki, Thessaloniki, GRC

**Keywords:** case report, median nerve, hematoma, epineurium, carpal tunnel syndrome

## Abstract

Carpal tunnel syndrome (CTS) is a common neuropathy with various underlying causes, posing diagnostic and management challenges for healthcare providers. The condition is typically associated with repetitive strain, idiopathic factors, or anatomical variations, leading to the compression of the median nerve within the carpal tunnel. We describe a case of a 46-year-old male who presented with recurrent CTS symptoms one year after a successful carpal tunnel release surgery. The symptoms resurfaced following a minor wrist trauma, leading to pain, numbness, and hand weakness. Despite initial conservative management, including immobilization and NSAIDs, the symptoms persisted. Further investigation and exploratory surgery revealed a rare subepineural hematoma of the median nerve, which was subsequently drained, resulting in immediate and lasting symptom relief. This case demonstrates the importance of considering uncommon etiologies such as subepineural hematomas in patients with recurrent CTS and underscores the need for a thorough diagnostic approach to ensure effective treatment.

## Introduction

Carpal tunnel syndrome (CTS) is a prevalent condition characterized by the entrapment and compression of the median nerve as it traverses through the carpal tunnel, a narrow passageway in the wrist [1]. This syndrome is the most common type of peripheral nerve entrapment neuropathy, particularly at the wrist level, affecting a significant portion of the population [1]. Epidemiological studies suggest that approximately one in 10 individuals may develop CTS at some point in their lives, with a notable gender disparity; women are approximately six times more likely to be affected than men, particularly between the ages of 65 and 74 years [2]. The hallmark symptoms of CTS include tingling, numbness, and pain in the thumb, index, middle, and radial half of the ring finger, which can progress to hand weakness and muscle atrophy if left untreated. While these symptoms are commonly seen in CTS, their occurrence without the typical presentation of CTS is relatively rare [3].

Understanding the pathophysiology of CTS is crucial for its management. The median nerve, which originates from the C5-T1 spinal nerves and traverses through the brachial plexus, is vital for both sensory and motor functions of the hand. Increased pressure within the confined space of the carpal tunnel, whether due to inflammation, repetitive motions, or trauma-induced hematomas, can lead to ischemia and subsequent disruption of nerve conduction, resulting in the classical symptoms of CTS [4]. In this case report, we present a rare case of CTS due to a subepineural hematoma of the median nerve following wrist trauma in a patient with a history of previous carpal tunnel release surgery.

## Case presentation

A 46-year-old Caucasian male presented to our private practice with complaints of pain radiating to the right arm, predominantly during night hours. The patient also reported hand weakness and decreased sensation along the distribution of the median nerve. His medical history was significant for a carpal tunnel release surgery performed one year prior, which had initially resulted in near-complete symptom resolution. However, he returned to our practice with a recurrence of symptoms that had progressively worsened over the past several weeks.

On physical examination, the patient exhibited decreased sensation throughout the median nerve distribution on the volar aspect of the hand. There was also a noted decrease in the strength of the abductor pollicis brevis muscle. Provocative testing was positive for both Tinel's sign and Phalen's test, suggesting irritation of the median nerve [5]. Given the clinical presentation, a nerve conduction velocity (NCV) study was conducted, which confirmed the diagnosis of CTS with significant motor and sensory latency. Based on these findings, the patient was scheduled for carpal tunnel release surgery the following day.

The surgical procedure involved a standard carpal tunnel release through a 3 cm longitudinal incision on the palmar side of the wrist, extending from the wrist flexion crease to Kaplan's cardinal line. The flexor tendon retinaculum was identified and incised longitudinally, carefully preserving the median nerve. The carpal tunnel was opened, and the median nerve was inspected to ensure there was no severe compression. Postoperatively, the patient's symptoms improved markedly, with almost complete relief from pain and restoration of sensation by the first postoperative day. At the one-month follow-up, the patient was asymptomatic and had resumed his normal activities without any limitations.

**Figure 1:**
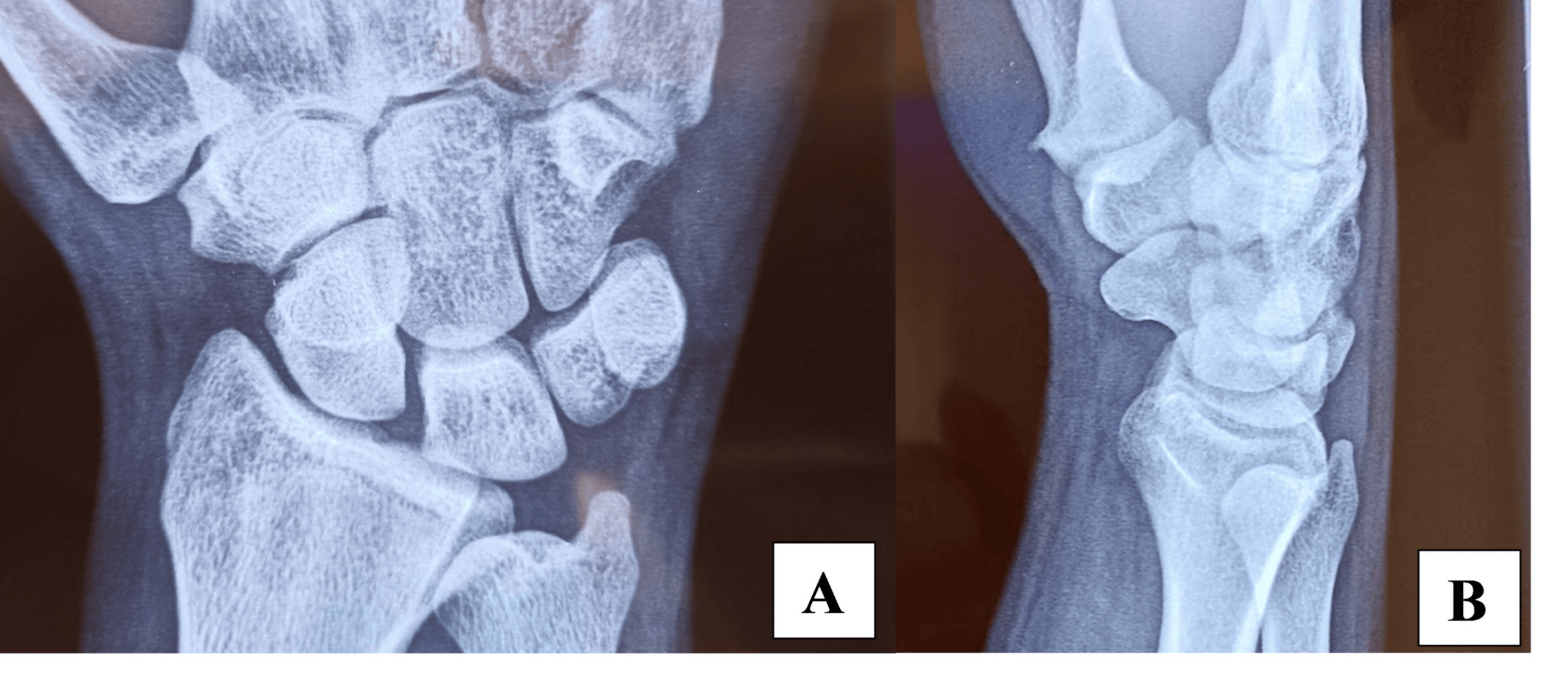
X-ray imaging of the wrist in anteroposterior (A) and lateral (B) views, showing normal bone alignment with no fractures or dislocation.

One year later, the patient revisited our practice with a recurrence of similar symptoms. He reported a fall on his right wrist three days before the consultation, which he believed had triggered the recurrence. During the physical examination, there was no weakness in the thenar muscles, but the patient continued to exhibit decreased sensation in the median nerve distribution. Given the recurrence of symptoms and the patient’s recent trauma, imaging studies were promptly initiated. A plain radiograph (X-ray) of the wrist was performed to rule out fractures or significant bony abnormalities that might have resulted from the fall. The X-ray showed no fractures, dislocations, or obvious bone pathology (Figure 1). Subsequently, an ultrasound of the wrist was conducted to assess soft tissue structures, revealing signs of edema around the carpal tunnel but no clear evidence of a compressive mass or structural deformities.

Given the persistence of symptoms, a conservative treatment approach was initially recommended. The patient was prescribed nonsteroidal anti-inflammatory drugs (NSAIDs) and his wrist was immobilized with a splint for 10 days to reduce inflammation and prevent further trauma. Following the removal of the cast, the patient underwent a two-week physical therapy program aimed at improving wrist mobility and strength. Despite these interventions, the patient did not experience significant improvement, prompting a repeat NCV study. The study once again demonstrated motor and sensory latency of the right median nerve, indicative of ongoing compression. After two months post-trauma, we performed the surgery.

**Figure 2:**
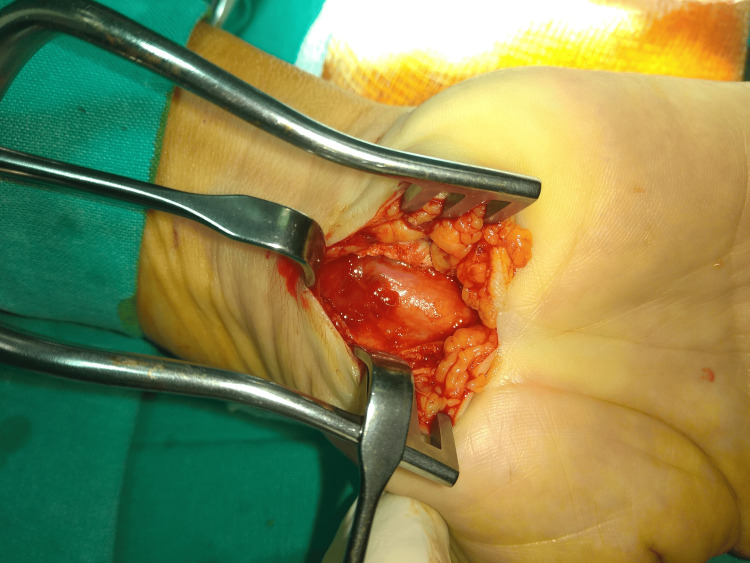
Intraoperative view of the subepineural hematoma compressing the median nerve within the carpal tunnel.

Due to the lack of improvement with conservative management, exploratory surgery was performed under local anesthesia without tourniquet control (Figure 2).

A 3 cm longitudinal incision was made on the palmar side of the wrist in the interthenar region, similar to the previous surgical approach. The flexor tendon retinaculum was identified and incised longitudinally, just as in the first procedure, ensuring the preservation of the median nerve. Upon exploration, the median nerve was found to be under mild compression by a subepineural hematoma. A longitudinal incision was made to expose and relieve the pressure on the median nerve. No tissue samples were sent for histopathological examination, as the focus was on decompressing the nerve by draining the hematoma and directly visualizing the nerve fascicles. The incision was closed and the wrist was bandaged.

Postoperatively, the patient reported immediate relief from his symptoms, with significant improvement noted from the first night after surgery. At a two-month follow-up, the patient had returned to his previous activities without any recurrence of symptoms. By the end of a six-month follow-up period, the patient remained asymptomatic, fully resuming his daily and professional activities. In adherence to ethical guidelines, written informed consent was obtained from the patient prior to all procedures and for the publication of this case report. The patient was fully informed about the nature of the case, the treatments administered, and the potential outcomes, and he agreed to the publication of his medical information for educational purposes.

## Discussion

CTS is a prevalent neuropathy characterized by the compression of the median nerve as it traverses through the carpal tunnel, leading to symptoms such as pain, numbness, and tingling in the hand [6]. In this report, we presented a rare and unique case of CTS caused by a subepineural hematoma of the median nerve following wrist trauma in a patient who had previously undergone carpal tunnel release surgery. This case is particularly noteworthy due to the identification of a subepineural hematoma - a highly unusual and scarcely documented cause of recurrent CTS symptoms. The diagnosis of the subepineural hematoma was facilitated using magnifying loupes during surgery, which allowed for a detailed examination of the nerve structures. After incising the paraneurium and epineurium, the hematoma was identified as being located between the median nerve and its surrounding connective tissue sheath. This differentiates subepineural hematomas, which form outside the nerve itself, from intraneural hematomas that occur within the nerve tissue [6]. Unlike the more common etiologies of CTS, such as repetitive strain, idiopathic factors, or structural anomalies, our patient's symptoms recurred due to the formation of a hematoma within the median nerve sheath after a minor wrist injury. This hematoma exerted enough pressure on the median nerve to reproduce the classic symptoms of CTS, despite prior successful surgical intervention. Initial conservative treatments, including immobilization and NSAIDs, failed to alleviate the symptoms, leading to the decision to perform exploratory surgery. During surgery, the hematoma was identified and successfully drained, resulting in immediate and lasting relief of the symptoms.

CTS is a neuropathy caused by the entrapment and continuous compression of the median nerve as it passes through the carpal tunnel, a structure located on the palm side of the wrist [6]. This tunnel is formed by the carpal bones and the flexor retinaculum. The median nerve, which provides sensory function to the thumb, index, middle, and radial half of the ring fingers and motor function to the muscles controlling finger movement, is responsible for the intricate movements of the hand [7]. Specifically, the nine flexor tendons associated with the median nerve play a crucial role in hand function: four flexor digitorum superficialis tendons flex the middle phalanges, four flexor digitorum profundus tendons flex the distal phalanges, and the flexor pollicis longus tendon flexes the distal phalanx of the thumb. When the median nerve is compressed, it disrupts normal nerve function, leading to the hallmark symptoms of CTS [7].

The occurrence of CTS is typically due to increased pressure within the carpal tunnel, which strains the median nerve [1]. This nerve originates from the spinal nerves C5-T1 and is one of the three main nerves that supply the muscles of the forearm and hand [4,8]. It plays a crucial role in transmitting sensory and motor signals throughout the hand. A key factor in maintaining proper nerve function is the blood supply, provided by small arteries and veins known as the vasa nervorum [9]. The high metabolic demands of peripheral nerves necessitate a continuous supply of oxygen and nutrients, as well as effective removal of metabolic waste [9]. Conditions that increase pressure in the carpal tunnel, such as inflammation from repetitive motions, fluid retention due to medical conditions like rheumatoid arthritis or hypothyroidism, or trauma-induced hematomas, can lead to median nerve ischemia, disrupting normal nerve conduction and causing demyelination and fibrosis over time [10].

The occurrence of subepineural hematomas as a cause of CTS is exceedingly rare in the literature [11-13]. Nerve hematomas can sometimes occur spontaneously, especially in the presence of factors such as blood clotting disorders, vascular abnormalities, or the use of certain medications, particularly anticoagulants [11]. Advanced age also increases the risk of spontaneous bleeding [13]. However, trauma remains the primary cause of hematoma formation, as seen in our case [6]. The median artery, found in approximately 10% of the population, runs alongside the median nerve in the forearm, between the radial and ulnar arteries [7]. This artery can contribute to hematoma development due to its proximity to the nerve [7]. In our case, the hematoma was located beneath the paraneurium and epineurium, highlighting the involvement of these structures in the pathophysiology of CTS. Typically, recurrent or persistent CTS symptoms post-surgery are due to factors such as scar tissue formation, incomplete release of the transverse carpal ligament, or nerve damage from the initial procedure [14]. Much of the existing literature focuses on other types of nerve compressions or anatomical abnormalities that present similarly [12,15]. Aside from subepineural hematomas, other rare anatomical variations and pathological conditions can lead to CTS. These include the presence of anomalous muscles, such as additional or enlarged muscles within the carpal tunnel, and a persistent median artery, an embryological remnant that can compress the nerve [7,16]. More commonly, CTS is associated with mass lesions such as ganglion cysts, synovial cysts, and lipomas, soft tissue tumors that develop within the carpal tunnel and exert pressure on the median nerve [17-19]. Although these variations and lesions are less common, they should be considered in the differential diagnosis of CTS, particularly in patients who do not respond to standard treatments.

The symptoms of CTS include paresthesia (tingling or numbness) in the fingers, pain that often radiates from the arm down into the hand, particularly worsening at night or during activities involving wrist flexion, hand weakness, and atrophy of the thenar muscles [20]. Diagnosis of CTS is typically confirmed through a combination of clinical tests, including Tinel’s sign and Phalen’s maneuver, as well as nerve conduction studies and ultrasonography [5]. These tools help to evaluate the extent of nerve compression and assess the functional impairment of the median nerve.

From a clinical management perspective, treatment for CTS varies depending on the severity of the condition. Although there is a possibility for nerve hematomas to resolve spontaneously, especially in cases involving factors like anticoagulant use, vascular abnormalities, or increased patient age, trauma remains a significant cause of hematoma formation [4]. In this case, the decision to proceed with surgery was based on the persistence of symptoms and the lack of improvement with conservative measures [4]. Conservative treatment options include ergonomic modifications to reduce strain on the wrist, the use of splints to maintain a neutral hand position, and physical therapy aimed at improving wrist strength and mobility while minimizing pressure on the median nerve [1]. In more advanced cases, surgical intervention, such as carpal tunnel release, may be necessary [4,8]. This procedure can be performed either endoscopically or through open surgery to relieve pressure on the median nerve. It is also important to consider rarer causes of CTS, such as anatomical variations or mass lesions within the carpal tunnel, which can contribute to nerve compression [6]. In cases like ours, where a rare etiology such as a subepineural hematoma is involved, surgical exploration becomes essential not only for diagnosis but also for therapeutic intervention. The success of the surgical drainage of the hematoma in our patient’s case underscores the importance of individualized treatment approaches, particularly in atypical or recurrent CTS cases.

Our experience highlights the importance of considering rare causes, such as subepineural hematoma, in the differential diagnosis of recurrent CTS. While conservative treatment should be the initial approach, this case underscores the need to remain vigilant for uncommon etiologies that may require surgical intervention when standard treatments do not provide relief.

## Conclusions

In conclusion, this case underscores the significance of including rare etiologies like subepineural hematomas in the diagnostic considerations for recurrent CTS, particularly in patients with a history of wrist trauma or prior surgical procedures. The successful identification and management of the hematoma through surgical exploration and drainage led to immediate and lasting symptom relief, underscoring the critical role of thorough diagnostic evaluation and personalized treatment in such atypical cases. Future studies should focus on further exploring the incidence, diagnosis, and optimal management strategies for trauma-induced subepineural hematomas to improve outcomes in similar cases. This case contributes to the limited body of knowledge on trauma-induced CTS and emphasizes the need for heightened clinical awareness and a comprehensive approach to managing recurrent or complex presentations of CTS.
